# Supplementary material for: Foliar Fungal Endophytes in a Tree Diversity Experiment Are Driven by the Identity but Not the Diversity of Tree Species
Source: Life (Basel). 2021 Oct 13;11(10):1081. doi: 10.3390/life11101081 (PMC8539603; doi:10.3390/life11101081)
Supplement: Supplementary file 1 [file life-11-01081-s001.zip › Figure S1.pdf]

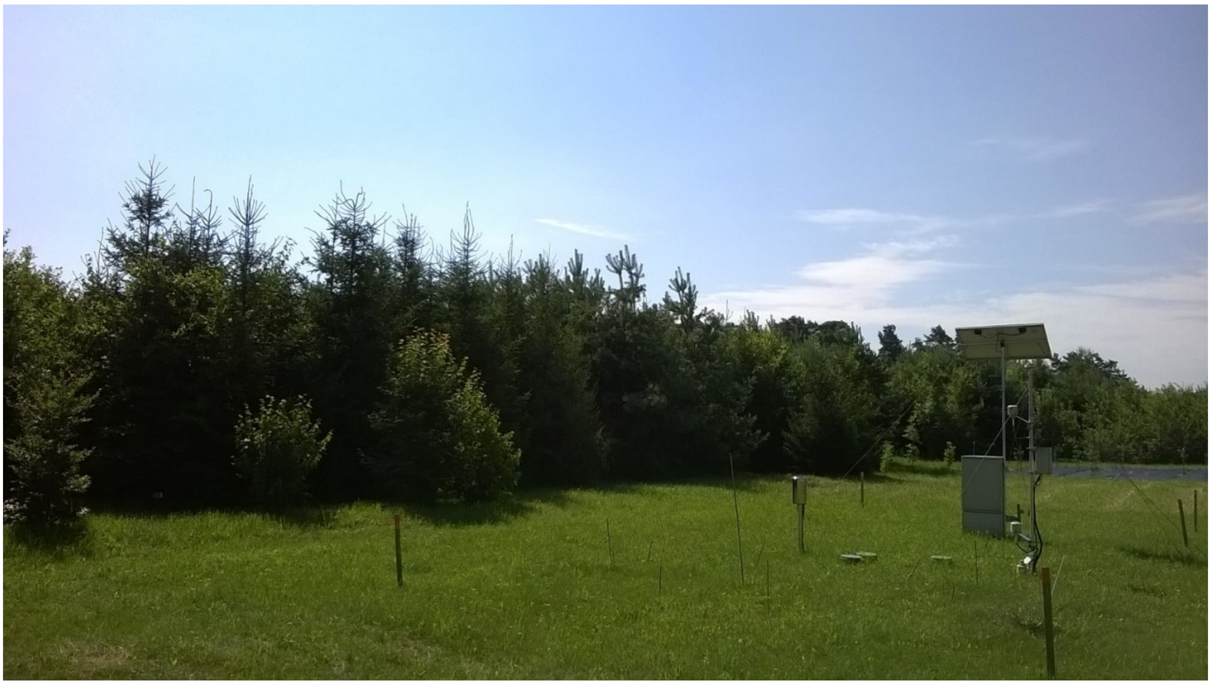

Figure S1. Photo of the densely vegetated plots at the Kreinitz tree diversity experiment in 2016 (This study was conducted one year later, in 2017).
