# Supplementary material for: Foliar Fungal Endophytes in a Tree Diversity Experiment Are Driven by the Identity but Not the Diversity of Tree Species
Source: Life (Basel). 2021 Oct 13;11(10):1081. doi: 10.3390/life11101081 (PMC8539603; doi:10.3390/life11101081)
Supplement: Supplementary file 1 [file life-11-01081-s001.zip › Table S2.pdf]

Table S2. Endophytic OTUs collected from leaf samples of beech (*Fagus sylvatica*), oak (*Quercus petraea*) and spruce (*Pinus sylvestris*) at the Kreinitz tree diversity experiment (www.treedivnet.ugent.be/ExpKreinitz.html) in 2017.

OTU_ID	Phylum	Class	Order	Family	Genus	Species	In beech samples	In oak samples	In spruce samples
OTU_001	Ascomycota	Saccharomycetes	Saccharomycetales	Dipodascaceae	unidentified	unidentified	TRUE	TRUE	TRUE
OTU_002	Ascomycota	Saccharomycetes	Saccharomycetales	Dipodascaceae	Dipodascus	Dipodascus_geotrichum	TRUE	TRUE	TRUE
OTU_003	Basidiomycota	Malasseziomycetes	Malasseziales	Malasseziaceae	Malassezia	Malassezia_restricta	TRUE	TRUE	TRUE
OTU_004	Basidiomycota	Malasseziomycetes	Malasseziales	Malasseziaceae	Malassezia	Malassezia_restricta	TRUE	TRUE	TRUE
OTU_005	Ascomycota	Saccharomycetes	Saccharomycetales	Saccharomycetales_fam_Incertae_sedis	unidentified	unidentified	TRUE	TRUE	TRUE
OTU_006	Basidiomycota	Malasseziomycetes	Malasseziales	unidentified	unidentified	unidentified	TRUE	TRUE	TRUE
OTU_007	Ascomycota	Leotiomycetes	Erysiphales	Erysiphaceae	Erysiphe	Erysiphe_sp		TRUE	TRUE
OTU_008	Ascomycota	Saccharomycetes	Saccharomycetales	Metschnikowiaceae	Clavispora	Clavispora_lusitaniae	TRUE	TRUE	TRUE
OTU_009	Basidiomycota	Malasseziomycetes	Malasseziales	Malasseziaceae	Malassezia	unidentified	TRUE	TRUE	TRUE
OTU_010	Ascomycota	Leotiomycetes	Helotiales	unidentified	unidentified	unidentified	TRUE	TRUE	TRUE
OTU_011	Ascomycota	Sordariomycetes	Xylariales	Amphisphaeriaceae	unidentified	unidentified	TRUE	TRUE	TRUE
OTU_012	Basidiomycota	Malasseziomycetes	Malasseziales	Malasseziaceae	Malassezia	Malassezia_globosa	TRUE	TRUE	TRUE
OTU_013	Basidiomycota	Microbotryomycetes	Sporidiobolales	Sporidiobolaceae	Rhodotorula	Rhodotorula_mucilaginosa	TRUE	TRUE	TRUE
OTU_014	Ascomycota	Eurotiomycetes	Eurotiales	Aspergillaceae	Penicillium	Penicillium_roqueforti	TRUE	TRUE	TRUE
OTU_015	Basidiomycota	Exobasidiomycetes	Entylomatales	Entylomatales_fam_Incertae_sedis	Tilletiopsis	Tilletiopsis_washingtonensis	TRUE	TRUE	TRUE
OTU_016	Ascomycota	Dothideomycetes	Capnodiales	Mycosphaerellaceae	Mycosphaerella	Mycosphaerella_tassiana	TRUE	TRUE	TRUE
OTU_017	Ascomycota	Dothideomycetes	Capnodiales	Mycosphaerellaceae	Ramularia	Ramularia_unterseheri	TRUE	TRUE	TRUE
OTU_018	Basidiomycota	Tremellomycetes	Cystofilobasidiales	Mrakiaceae	Itersonilia	Itersonilia_perplexans	TRUE	TRUE	TRUE
OTU_019	Ascomycota	Saccharomycetes	Saccharomycetales	Metschnikowiaceae	Clavispora	unidentified	TRUE	TRUE	TRUE
OTU_020	Ascomycota	Saccharomycetes	Saccharomycetales	Debaryomycetaceae	Debaryomyces	Debaryomyces_hansenii	TRUE	TRUE	TRUE
OTU_021	unidentified	unidentified	unidentified	unidentified	unidentified	unidentified	TRUE		
OTU_022	Ascomycota	Leotiomycetes	Helotiales	unidentified	unidentified	unidentified	TRUE	TRUE	TRUE
OTU_023	Basidiomycota	Agaricomycetes	Agaricales	Tricholomataceae	Clitocybe	Clitocybe_nebularis			TRUE
OTU_024	Ascomycota	unidentified	unidentified	unidentified	unidentified	unidentified			TRUE

OTU_025	Ascomycota	Eurotiomycetes	Eurotiales	Aspergillaceae	Penicillium	Penicillium_thymicola	TRUE	TRUE	TRUE
OTU_026	Basidiomycota	Exobasidiomycetes	Entylomatales	unidentified	unidentified	unidentified	TRUE	TRUE	TRUE
OTU_027	Ascomycota	Saccharomycetes	Saccharomycetales	Metschnikowiaceae	Metschnikowia	Metschnikowia_viticola	TRUE	TRUE	TRUE
OTU_028	Basidiomycota	Agaricomycetes	Russulales	Russulaceae	Russula	Russula_cyanoxantha	TRUE	TRUE	TRUE
OTU_029	Basidiomycota	Microbotryomycetes	Sporidiobolales	Sporidiobolaceae	Sporobolomyces	Sporobolomyces_roseus	TRUE	TRUE	TRUE
OTU_030	Basidiomycota	Agaricomycetes	Atheliales	Atheliaceae	Tylospora	unidentified		TRUE	
OTU_031	Basidiomycota	Microbotryomycetes	Sporidiobolales	Sporidiobolaceae	Rhodotorula	Rhodotorula_diobovata	TRUE	TRUE	TRUE
OTU_032	Ascomycota	Leotiomycetes	Helotiales	Helotiales_fam_Incertae_sedis	Chalara	unidentified	TRUE	TRUE	TRUE
OTU_033	unidentified	unidentified	unidentified	unidentified	unidentified	unidentified	TRUE	TRUE	TRUE
OTU_034	Ascomycota	Leotiomycetes	Helotiales	Helotiaceae	Hymenoscyphus	Hymenoscyphus_caudatus	TRUE	TRUE	TRUE
OTU_035	Ascomycota	Dothideomycetes	Dothideomycetes_ord_Incertae_sedis	Nowamycetaceae	Nowamyces	Nowamyces_globulus	TRUE	TRUE	TRUE
OTU_036	Basidiomycota	Tremellomycetes	Tremellales	unidentified	unidentified	unidentified		TRUE	TRUE
OTU_037	Basidiomycota	Tremellomycetes	Tremellales	Bulleribasidiaceae	Vishniacozyma	Vishniacozyma_victoriae	TRUE	TRUE	TRUE
OTU_038	Basidiomycota	Tremellomycetes	Tremellales	Bulleribasidiaceae	Vishniacozyma	unidentified	TRUE	TRUE	TRUE
OTU_039	Ascomycota	Dothideomycetes	Capnodiales	Mycosphaerellaceae	Mycosphaerella	Mycosphaerella_flageoletiana	TRUE	TRUE	TRUE
OTU_040	Basidiomycota	Ustilaginomycetes	Ustilaginales	Ustilaginaceae	Pseudozyma	Pseudozyma_prolifica	TRUE	TRUE	TRUE
OTU_041	Ascomycota	Dothideomycetes	Capnodiales	Cladosporiaceae	Cladosporium	Cladosporium_delicatulum	TRUE	TRUE	TRUE
OTU_042	Ascomycota	Leotiomycetes	Helotiales	Hyaloscyphaceae	Austropezia	unidentified	TRUE	TRUE	TRUE
OTU_043	Ascomycota	Dothideomycetes	Capnodiales	unidentified	unidentified	unidentified	TRUE	TRUE	TRUE
OTU_044	Basidiomycota	Agaricomycetes	Agaricales	Clavariaceae	Clavaria	unidentified	TRUE	TRUE	TRUE
OTU_045	Basidiomycota	Malasseziomycetes	Malasseziales	Malasseziaceae	Malassezia	Malassezia_globosa	TRUE	TRUE	TRUE
OTU_046	Ascomycota	unidentified	unidentified	unidentified	unidentified	unidentified	TRUE	TRUE	
OTU_047	Basidiomycota	Agaricomycetes	Agaricales	Hygrophoraceae	Cuphophyllus	Cuphophyllus_pratensis	TRUE	TRUE	TRUE
OTU_048	Ascomycota	Sordariomycetes	Pleurotheciales	Pleurotheciaceae	Phaeoisaria	Phaeoisaria_loranthacearum		TRUE	
OTU_049	Ascomycota	Leotiomycetes	Helotiales	Leotiaceae	unidentified	unidentified		TRUE	TRUE
OTU_050	Basidiomycota	Cystobasidiomycetes	Erythrobasidiales	Erythrobasidiaceae	Erythrobasidium	Erythrobasidium_yunnanense	TRUE		
OTU_051	Rozellomycota	Rozellomycotina_cls_Incertae_sedis	GS11	unidentified	unidentified	unidentified			TRUE
OTU_052	Ascomycota	Leotiomycetes	Erysiphales	Erysiphaceae	Podosphaera	Podosphaera_leucotricha	TRUE	TRUE	TRUE

OTU_053	unidentified	unidentified	unidentified	unidentified	unidentified	unidentified	TRUE	TRUE	TRUE
OTU_054	Basidiomycota	Agaricomycetes	Agaricales	Tricholomataceae	Tricholoma	Tricholoma_umbonatum	TRUE		
OTU_055	Ascomycota	Dothideomycetes	Capnodiales	Mycosphaerellaceae	Pallidocercospora	Pallidocercospora_heimii	TRUE	TRUE	TRUE
OTU_056	Basidiomycota	Agaricomycetes	Agaricales	Hygrophoraceae	Hygrophorus	Hygrophorus_eburneus	TRUE	TRUE	
OTU_057	NA	NA	NA	NA	NA	No blast hit	TRUE	TRUE	
OTU_058	Basidiomycota	Tremellomycetes	Cystofilobasidiales	Cystofilobasidiaceae	Cystofilobasidium	Cystofilobasidium_ferigula	TRUE	TRUE	TRUE
OTU_059	Ascomycota	Dothideomycetes	Capnodiales	Mycosphaerellaceae	Mycosphaerella	Mycosphaerella_flageoletiana			TRUE
OTU_060	Ascomycota	Dothideomycetes	Capnodiales	Teratosphaeriaceae	Devriesia	Devriesia_pseudoamericana			TRUE
OTU_061	Ascomycota	Leotiomycetes	Helotiales	unidentified	unidentified	unidentified	TRUE		TRUE
OTU_062	Basidiomycota	Tremellomycetes	Filobasidiales	Filobasidiaceae	Filobasidium	Filobasidium_wieringae	TRUE	TRUE	TRUE
OTU_063	Basidiomycota	Agaricomycetes	Agaricales	Hygrophoraceae	Cuphophyllus	Cuphophyllus_pratensis	TRUE	TRUE	TRUE
OTU_064	Basidiomycota	Tremellomycetes	Filobasidiales	Filobasidiaceae	Naganishia	Naganishia_diffluens	TRUE	TRUE	TRUE
OTU_065	Ascomycota	Dothideomycetes	Pleosporales	Sporormiaceae	Preussia	unidentified	TRUE		
OTU_066	Ascomycota	Dothideomycetes	Pleosporales	Didymellaceae	Didymella	Didymella_rhei	TRUE	TRUE	TRUE
OTU_067	Ascomycota	Pezizomycetes	Pezizales	Pezizaceae	Hydnobolites	Hydnobolites_cerebriformis			TRUE
OTU_068	unidentified	unidentified	unidentified	unidentified	unidentified	unidentified	TRUE		
OTU_069	Ascomycota	Saccharomycetes	Saccharomycetales	Saccharomycetaceae	Issatchenkia	Issatchenkia_orientalis	TRUE	TRUE	TRUE
OTU_070	Basidiomycota	Agaricomycetes	Trechisporales	Hydnodontaceae	unidentified	unidentified	TRUE		
OTU_071	NA	NA	NA	NA	NA	No blast hit	TRUE	TRUE	
OTU_072	Ascomycota	unidentified	unidentified	unidentified	unidentified	unidentified	TRUE		
OTU_073	Ascomycota	Dothideomycetes	Capnodiales	Mycosphaerellaceae	unidentified	unidentified			TRUE
OTU_074	Ascomycota	Dothideomycetes	Pleosporales	Pleosporaceae	Alternaria	Alternaria_tenuissima	TRUE	TRUE	
OTU_075	Ascomycota	Leotiomycetes	Helotiales	Sclerotiniaceae	Botrytis	Botrytis_caroliniana		TRUE	
OTU_076	Basidiomycota	Agaricomycetes	Agaricales	Inocybaceae	Inocybe	Inocybe_assimilata		TRUE	
OTU_077	Ascomycota	Saccharomycetes	Saccharomycetales	Saccharomycodaceae	Hanseniaspora	Hanseniaspora_uvarum	TRUE	TRUE	TRUE
OTU_078	Ascomycota	Sordariomycetes	Sordariales	unidentified	unidentified	unidentified	TRUE	TRUE	
OTU_079	Basidiomycota	Microbotryomycetes	Microbotryales	Microbotryaceae	Microbotryum	Microbotryum_stellariae	TRUE	TRUE	TRUE
OTU_080	Ascomycota	Eurotiomycetes	Chaetothyriales	Herpotrichiellaceae	unidentified	unidentified			TRUE
OTU_081	Basidiomycota	Tremellomycetes	unidentified	unidentified	unidentified	unidentified		TRUE	

OTU_082	Ascomycota	Dothideomycetes	Pleosporales	unidentified	unidentified	unidentified	TRUE		
OTU_083	Ascomycota	Leotiomycetes	Helotiales	Hyaloscyphaceae	unidentified	unidentified	TRUE		
OTU_084	Ascomycota	Dothideomycetes	Capnodiales	unidentified	unidentified	unidentified	TRUE		
OTU_086	Ascomycota	Saccharomycetes	Saccharomycetales	Dipodascaceae	unidentified	unidentified		TRUE	
OTU_087	Basidiomycota	Agaricomycetes	Thelephorales	Thelephoraceae	Tomentella	unidentified	TRUE		
OTU_088	Basidiomycota	Tremellomycetes	Filobasidiales	Piskurozymaceae	Solicoccozyma	Solicoccozyma_terricola	TRUE	TRUE	TRUE
OTU_089	Basidiomycota	Exobasidiomycetes	Exobasidiales	Graphiolaceae	Graphiola	Graphiola_phoenicis	TRUE	TRUE	TRUE
OTU_090	Ascomycota	Dothideomycetes	Pleosporales	Amorosiaceae	Angustimassarina	Angustimassarina_acerina			TRUE
OTU_091	Rozellomycota	Rozellomycotina_cls_Incertae_sedis	GS11	unidentified	unidentified	unidentified		TRUE	
OTU_092	unidentified	unidentified	unidentified	unidentified	unidentified	unidentified		TRUE	
OTU_093	Ascomycota	Leotiomycetes	Helotiales	unidentified	unidentified	unidentified	TRUE	TRUE	TRUE
OTU_094	Basidiomycota	Cystobasidiomycetes	Cystobasidiomycetes_ord_Incertae_sedis	Symmetrosporaceae	Symmetrospora	unidentified	TRUE	TRUE	TRUE
OTU_095	Ascomycota	Dothideomycetes	Pleosporales	Didymellaceae	Neoascochyta	Neoascochyta_graminicola	TRUE	TRUE	TRUE
OTU_096	Ascomycota	Dothideomycetes	Pleosporales	Phaeosphaeriaceae	Setomelanomma	Setomelanomma_holmii			TRUE
OTU_097	Ascomycota	Dothideomycetes	Pleosporales	Phaeosphaeriaceae	Septoriella	Septoriella_phragmitis	TRUE		
OTU_098	Basidiomycota	Agaricomycetes	Thelephorales	Thelephoraceae	Tomentella	Tomentella_ellisii	TRUE		
OTU_100	Mortierellomycota	Mortierellomycetes	Mortierellales	Mortierellaceae	Mortierella	unidentified	TRUE		
OTU_101	Basidiomycota	Agaricomycetes	Russulales	Russulaceae	Russula	Russula_grisea		TRUE	
OTU_102	Ascomycota	Leotiomycetes	Helotiales	unidentified	unidentified	unidentified	TRUE	TRUE	TRUE
OTU_103	Ascomycota	Dothideomycetes	Capnodiales	Neodevriesiaceae	Neodevriesia	Neodevriesia_sexualis	TRUE		TRUE
OTU_104	NA	NA	NA	NA	NA	No blast hit		TRUE	
OTU_105	Mortierellomycota	Mortierellomycetes	Mortierellales	Mortierellaceae	Mortierella	Mortierella_longigemmata			TRUE
OTU_107	Basidiomycota	Tritirachiomycetes	Tritirachiales	Tritirachiaceae	Paratritirachium	unidentified	TRUE		
OTU_108	Ascomycota	Sordariomycetes	Chaetosphaeriales	Chaetosphaeriaceae	Chaetosphaeria	unidentified	TRUE		
OTU_110	Basidiomycota	Malasseziomycetes	Malasseziales	Malasseziaceae	Malassezia	Malassezia_globosa	TRUE	TRUE	TRUE
OTU_111	Ascomycota	Leotiomycetes	Helotiales	unidentified	unidentified	unidentified			TRUE
OTU_112	Ascomycota	Sordariomycetes	Xylariales	Xylariaceae	Nemania	Nemania_sp			TRUE
OTU_114	Basidiomycota	Tremellomycetes	Tremellales	Bulleraceae	Bullera	Bullera_crocea	TRUE	TRUE	TRUE

OTU_115	Ascomycota	Sordariomycetes	Hypocreales	Clavicipitaceae	Metarhizium	Metarhizium_anisopliae			TRUE
OTU_116	Ascomycota	Dothideomycetes	Pleosporales	Didymellaceae	Epicoccum	Epicoccum_dendrobii	TRUE		TRUE
OTU_117	Ascomycota	Eurotiomycetes	Eurotiales	Aspergillaceae	Penicillium	Penicillium_salamorum	TRUE		TRUE
OTU_118	Ascomycota	Dothideomycetes	Dothideales	Aureobasidiaceae	Kabatiella	Kabatiella_lini	TRUE	TRUE	TRUE
OTU_119	Ascomycota	Dothideomycetes	Pleosporales	Leptosphaeriaceae	Leptosphaeria	Leptosphaeria_rubefaciens	TRUE	TRUE	
OTU_120	Ascomycota	Saccharomycetes	Saccharomycetales	Saccharomycetaceae	Torulaspora	Torulaspora_delbrueckii	TRUE	TRUE	TRUE
OTU_121	Basidiomycota	Malasseziomycetes	Malasseziales	Malasseziaceae	Malassezia	Malassezia_globosa	TRUE	TRUE	TRUE
OTU_122	Ascomycota	Saccharomycetes	Saccharomycetales	Dipodascaceae	unidentified	unidentified	TRUE	TRUE	TRUE
OTU_123	Ascomycota	Leotiomycetes	Helotiales	Myxotrichaceae	Oidiodendron	Oidiodendron_rhodogenum	TRUE		
OTU_124	Ascomycota	unidentified	unidentified	unidentified	unidentified	unidentified	TRUE		TRUE
OTU_125	Ascomycota	Dothideomycetes	Venturiales	Venturiaceae	unidentified	unidentified			TRUE
OTU_126	Ascomycota	Eurotiomycetes	Eurotiales	Aspergillaceae	Penicillium	Penicillium_penicillioides	TRUE		
OTU_127	Basidiomycota	Tremellomycetes	Tremellales	Bulleribasidiaceae	Dioszegia	Dioszegia_hungarica	TRUE	TRUE	TRUE
OTU_128	Basidiomycota	Cystobasidiomycetes	Cystobasidiomycetes_ord_Incertae_sedis	Symmetrosporaceae	Symmetrospora	unidentified	TRUE		TRUE
OTU_129	Ascomycota	Sordariomycetes	Sordariales	Lasiosphaeriaceae	Apodus	Apodus_deciduus			TRUE
OTU_130	Ascomycota	Dothideomycetes	Capnodiales	Mycosphaerellaceae	Phaeophleospora	Phaeophleospora_stramenti			TRUE
OTU_131	Rozellomycota	Rozellomycotina_cls_Incertae_sedis	GS11	unidentified	unidentified	unidentified			TRUE
OTU_132	Ascomycota	Dothideomycetes	Capnodiales	Cladosporiaceae	Cladosporium	Cladosporium_sphaerospermum	TRUE		
OTU_133	Ascomycota	Sordariomycetes	Microascales	Microascaceae	Cephalotrichum	unidentified	TRUE		
OTU_134	Basidiomycota	Tremellomycetes	Tremellales	Bulleribasidiaceae	Vishniacozyma	Vishniacozyma_victoriae		TRUE	
OTU_135	Ascomycota	Saccharomycetes	Saccharomycetales	Phaffomycetaceae	Wickerhamomyces	Wickerhamomyces_anomalus	TRUE		
OTU_136	Ascomycota	Leotiomycetes	Rhytismatales	Rhytismataceae	Lophodermium	unidentified			TRUE
OTU_137	Basidiomycota	Agaricomycetes	Boletales	Melanogastraceae	Melanogaster	Melanogaster_broomeanus	TRUE		
OTU_138	Ascomycota	Lecanoromycetes	Teloschistales	Teloschistaceae	Xanthoria	Xanthoria_coomae			TRUE
OTU_139	Basidiomycota	Tremellomycetes	Tremellales	Syzygosporaceae	Syzygospora	Syzygospora_effibulata			TRUE
OTU_140	Basidiomycota	Agaricomycetes	Russulales	Russulaceae	Russula	Russula_puellaris		TRUE	
OTU_142	Basidiomycota	Tremellomycetes	Trichosporonales	Trichosporonaceae	Apiotrichum	Apiotrichum_porosum			TRUE
OTU_143	Ascomycota	Dothideomycetes	Capnodiales	Dissoconiaceae	Uwebraunia	Uwebraunia_dekkeri	TRUE		TRUE

OTU_145	NA	NA	NA	NA	NA	No blast hit		TRUE	TRUE
OTU_146	Basidiomycota	Tremellomycetes	Tremellales	Bulleribasidiaceae	Vishniacozyma	Vishniacozyma_carnescens	TRUE	TRUE	
OTU_147	Mucoromycota	Mucoromycetes	Mucorales	Mucoraceae	Mucor	Mucor_bainieri	TRUE		TRUE
OTU_148	unidentified	unidentified	unidentified	unidentified	unidentified	unidentified	TRUE	TRUE	
OTU_149	Ascomycota	Leotiomycetes	Thelebolales	Pseudeurotiaceae	Leuconeurospora	unidentified		TRUE	
OTU_150	Ascomycota	unidentified	unidentified	unidentified	unidentified	unidentified			TRUE
OTU_151	Ascomycota	Sordariomycetes	Sordariales	Lasiosphaeriaceae	unidentified	unidentified	TRUE	TRUE	
OTU_152	Ascomycota	Dothideomycetes	Dothideales	Dothioraceae	Hormonema	unidentified	TRUE		
OTU_153	Mortierellomycota	Mortierellomycetes	Mortierellales	Mortierellaceae	Mortierella	Mortierella_macrocystis		TRUE	TRUE
OTU_154	Ascomycota	Saccharomycetes	Saccharomycetales	Saccharomycetaceae	Kluyveromyces	Kluyveromyces_lactis			TRUE
OTU_155	Ascomycota	Dothideomycetes	Capnodiales	Mycosphaerellaceae	unidentified	unidentified			TRUE
OTU_158	Ascomycota	Sordariomycetes	Hypocreales	Hypocreaceae	Trichoderma	Trichoderma_aureoviride		TRUE	
OTU_159	Basidiomycota	Tremellomycetes	Tremellales	Rhynchogastremataceae	Papiliotrema	Papiliotrema_japonica	TRUE		TRUE
OTU_160	Basidiomycota	Tremellomycetes	Tremellales	Bulleribasidiaceae	Hannaella	Hannaella_kunmingensis	TRUE		
OTU_161	Basidiomycota	Tremellomycetes	Tremellales	unidentified	unidentified	unidentified	TRUE		TRUE
OTU_162	Ascomycota	Dothideomycetes	unidentified	unidentified	unidentified	unidentified	TRUE		TRUE
OTU_163	Ascomycota	Dothideomycetes	Capnodiales	unidentified	unidentified	unidentified			TRUE
OTU_164	Ascomycota	Eurotiomycetes	Eurotiales	Aspergillaceae	Penicillium	Penicillium_bilaiae			TRUE
OTU_165	Mortierellomycota	Mortierellomycetes	Mortierellales	Mortierellaceae	Mortierella	Mortierella_basiparvispora			TRUE
OTU_166	Ascomycota	Leotiomycetes	Thelebolales	Pseudeurotiaceae	unidentified	unidentified	TRUE		
OTU_167	Ascomycota	Lichinomycetes	Lichinales	Lichinaceae	Phylliscum	Phylliscum_demangeonii		TRUE	
OTU_168	Basidiomycota	Agaricomycetes	unidentified	unidentified	unidentified	unidentified		TRUE	
OTU_169	Ascomycota	Saccharomycetes	Saccharomycetales	Metschnikowiaceae	Clavispora	unidentified	TRUE	TRUE	TRUE
OTU_170	Basidiomycota	Microbotryomycetes	Sporidiobolales	Sporidiobolaceae	Sporobolomyces	Sporobolomyces_oryzicola	TRUE	TRUE	TRUE
OTU_172	Basidiomycota	Malasseziomycetes	Malasseziales	Malasseziaceae	Malassezia	Malassezia_globosa	TRUE	TRUE	
OTU_173	Ascomycota	Sordariomycetes	Coniochaetales	Coniochaetaceae	Coniochaeta	Coniochaeta_lignicola	TRUE	TRUE	TRUE
OTU_174	Basidiomycota	Tremellomycetes	Filobasidiales	Filobasidiaceae	Filobasidium	Filobasidium_stepposum	TRUE		
OTU_175	Basidiomycota	Malasseziomycetes	Malasseziales	Malasseziaceae	Malassezia	Malassezia_arunalokei	TRUE		TRUE
OTU_176	Basidiomycota	Agaricomycetes	Russulales	Russulaceae	Russula	Russula_nigricans			TRUE

OTU_177	unidentified	unidentified	unidentified	unidentified	unidentified	unidentified			TRUE
OTU_178	Basidiomycota	Agaricomycetes	Sebacinales	Sebacinaceae	Sebacina	unidentified			TRUE
OTU_179	Ascomycota	Leotiomycetes	Helotiales	unidentified	unidentified	unidentified	TRUE		
OTU_180	Ascomycota	Sordariomycetes	Hypocreales	Nectriaceae	unidentified	unidentified	TRUE		
OTU_181	Mortierellomycota	Mortierellomycetes	Mortierellales	Mortierellaceae	Mortierella	Mortierella_horticola	TRUE		
OTU_182	Rozellomycota	unidentified	unidentified	unidentified	unidentified	unidentified	TRUE		
OTU_183	Ascomycota	Saccharomycetes	Saccharomycetales	Phaffomycetaceae	Wickerhamomyces	Wickerhamomyces_anomalus		TRUE	
OTU_185	Basidiomycota	Agaricomycetes	Agaricales	Hygrophoraceae	Hygrophorus	Hygrophorus_discoxanthus		TRUE	
OTU_186	NA	NA	NA	NA	NA	No blast hit			TRUE
OTU_187	Basidiomycota	Agaricomycetes	Agaricales	Amanitaceae	Amanita	Amanita_submembranacea	TRUE		
OTU_188	Ascomycota	Eurotiomycetes	Eurotiales	Aspergillaceae	Penicillium	Penicillium_jensenii	TRUE		
OTU_190	Ascomycota	Saccharomycetes	Saccharomycetales	Saccharomycetales_fam_Incertae_sedis	Candida	Candida_vrieseae			TRUE
OTU_191	Ascomycota	Dothideomycetes	Dothideales	Dothioraceae	Dothiora	Dothiora_prunorum		TRUE	
OTU_192	Basidiomycota	Agaricomycetes	Russulales	Russulaceae	Russula	unidentified	TRUE		TRUE
OTU_193	unidentified	unidentified	unidentified	unidentified	unidentified	unidentified	TRUE		
OTU_194	Ascomycota	Dothideomycetes	Pleosporales	Phaeosphaeriaceae	Setomelanomma	Setomelanomma_holmii	TRUE		
OTU_195	Ascomycota	Dothideomycetes	Pleosporales	Phaeosphaeriaceae	Setomelanomma	unidentified			TRUE
OTU_196	Basidiomycota	Agaricomycetes	Corticiales	Vuilleminiaceae	Vuilleminia	Vuilleminia_comedens	TRUE		
OTU_197	Basidiomycota	Ustilaginomycetes	Ustilaginales	Ustilaginaceae	Macalpinomyces	Macalpinomyces_tristachyae	TRUE		TRUE
OTU_198	Ascomycota	Dothideomycetes	Dothideales	Aureobasidiaceae	Aureobasidium	Aureobasidium_thailandense	TRUE		
OTU_199	Ascomycota	Dothideomycetes	Pleosporales	unidentified	unidentified	unidentified	TRUE		
OTU_201	Basidiomycota	Agaricomycetes	Russulales	Russulaceae	Russula	unidentified	TRUE		
OTU_202	Basidiomycota	Cystobasidiomycetes	Erythrobasidiales	Erythrobasidiales_fam_Incertae_sedis	Sakaguchia	Sakaguchia_dacryoidea	TRUE		
OTU_203	Ascomycota	Sordariomycetes	Hypocreales	Clavicipitaceae	Metapochonia	Metapochonia_goniodes			TRUE
OTU_204	Ascomycota	Dothideomycetes	Pleosporales	unidentified	unidentified	unidentified	TRUE		
OTU_205	Basidiomycota	Agaricomycetes	Boletales	Boletaceae	Xerocomellus	Xerocomellus_porosporus	TRUE		
OTU_206	Basidiomycota	Exobasidiomycetes	Entylomatales	unidentified	unidentified	unidentified		TRUE	
OTU_207	Ascomycota	Dothideomycetes	Pleosporales	Pleosporaceae	Alternaria	Alternaria_infectoria	TRUE		

OTU_208	Ascomycota	Eurotiomycetes	Eurotiales	Aspergillaceae	Penicillium	Penicillium_sumatraense		TRUE	
OTU_209	Basidiomycota	Agaricomycetes	unidentified	unidentified	unidentified	unidentified			TRUE
OTU_210	Mortierellomycota	Mortierellomycetes	Mortierellales	Mortierellaceae	Mortierella	unidentified			TRUE
OTU_211	Basidiomycota	Agaricomycetes	Corticiales	Corticiaceae	Corticium	Corticium_confine	TRUE		
OTU_214	Basidiomycota	Agaricomycetes	Hymenochaetales	unidentified	unidentified	unidentified	TRUE		
OTU_216	Basidiomycota	Tremellomycetes	Tremellales	Naemateliaceae	Dimennazyma	Dimennazyma_cistialbidi	TRUE		
OTU_217	Basidiomycota	Tremellomycetes	Tremellales	Bulleraceae	Genolevuria	Genolevuria_amylolytica			TRUE
OTU_218	Ascomycota	Dothideomycetes	Pleosporales	Pleosporales_fam_Incertae_sedis	Pseudodidymella	Pseudodidymella_minima		TRUE	TRUE
OTU_219	Ascomycota	Leotiomycetes	Helotiales	unidentified	unidentified	unidentified		TRUE	
OTU_221	Basidiomycota	Tremellomycetes	Tremellales	Tremellaceae	Cryptococcus	unidentified		TRUE	
OTU_222	Ascomycota	Leotiomycetes	Helotiales	unidentified	unidentified	unidentified			TRUE
OTU_223	Basidiomycota	Agaricomycetes	Boletales	Serpulaceae	Serpula	Serpula_himantioides			TRUE
OTU_224	NA	NA	NA	NA	NA	No blast hit	TRUE		
OTU_227	Basidiomycota	Agaricomycetes	Agaricales	Cortinariaceae	Cortinarius	Cortinarius_anthracinus			TRUE
OTU_228	Rozellomycota	unidentified	unidentified	unidentified	unidentified	unidentified			TRUE
OTU_229	Basidiomycota	Tremellomycetes	Tremellales	Tremellaceae	Tremella	unidentified		TRUE	
OTU_230	Basidiomycota	Tremellomycetes	Filobasidiales	Filobasidiaceae	Filobasidium	Filobasidium_floriforme		TRUE	
OTU_231	Ascomycota	Saccharomycetes	Saccharomycetales	Phaffomycetaceae	Cyberlindnera	Cyberlindnera_jadinii			TRUE
OTU_232	Basidiomycota	Tremellomycetes	Cystofilobasidiales	Mrakiaceae	Mrakia	Mrakia_blollopis			TRUE
OTU_234	unidentified	unidentified	unidentified	unidentified	unidentified	unidentified			TRUE
OTU_235	Ascomycota	Dothideomycetes	Capnodiales	unidentified	unidentified	unidentified	TRUE		TRUE
OTU_237	Ascomycota	Dothideomycetes	Capnodiales	Mycosphaerellaceae	Phaeophleospora	Phaeophleospora_hymenocallidicola		TRUE	
OTU_238	Ascomycota	Sordariomycetes	Hypocreales	Nectriaceae	Neonectria	Neonectria_lugdunensis	TRUE		
OTU_239	Basidiomycota	Tremellomycetes	Tremellales	Tremellaceae	Cryptococcus	unidentified			TRUE
OTU_240	Ascomycota	Sordariomycetes	Hypocreales	Hypocreaceae	Trichoderma	Trichoderma_lacuwombatense		TRUE	
OTU_241	Ascomycota	Saccharomycetes	Saccharomycetales	unidentified	unidentified	unidentified		TRUE	
OTU_243	Ascomycota	Eurotiomycetes	Verrucariales	Verrucariaceae	Atla	Atla_vitikainenii	TRUE		
OTU_244	Basidiomycota	Agaricomycetes	Russulales	Russulaceae	Russula	Russula_cyanoxantha			TRUE
OTU_245	NA	NA	NA	NA	NA	No blast hit	TRUE	TRUE	

OTU_246	Ascomycota	Sordariomycetes	Coniochaetales	Coniochaetaceae	Coniochaeta	Coniochaeta_acaciae			TRUE
OTU_247	Ascomycota	Eurotiomycetes	Eurotiales	Aspergillaceae	Aspergillus	Aspergillus_penicillioides			TRUE
OTU_248	Ascomycota	Dothideomycetes	unidentified	unidentified	unidentified	unidentified			TRUE
OTU_249	NA	NA	NA	NA	NA	No blast hit	TRUE		
OTU_250	NA	NA	NA	NA	NA	No blast hit	TRUE		
OTU_251	Basidiomycota	Malasseziomycetes	Malasseziales	Malasseziaceae	Malassezia	Malassezia_globosa	TRUE		
OTU_252	Basidiomycota	Cystobasidiomycetes	Cystobasidiomycetes_ord_Incertae_sedis	Symmetrosporaceae	Symmetrospora	Symmetrospora_vermiculata	TRUE		
OTU_253	Ascomycota	Pezizomycetes	Pezizales	Pezizaceae	Peziza	Peziza_varia	TRUE	TRUE	
OTU_254	NA	NA	NA	NA	NA	No blast hit	TRUE		
OTU_255	NA	NA	NA	NA	NA	No blast hit	TRUE		
OTU_256	NA	NA	NA	NA	NA	No blast hit	TRUE		
